# The highways and byways of the brain

**DOI:** 10.1371/journal.pbio.3001612

**Published:** 2022-03-31

**Authors:** Claus C. Hilgetag, Basilis Zikopoulos

**Affiliations:** 1 Institute of Computational Neuroscience, University Medical Center Eppendorf, Hamburg University, Hamburg, Germany; 2 Department of Health Sciences, Boston University, Boston, Massachusetts, United States of America; 3 Department of Anatomy and Neurobiology, Boston University School of Medicine, Boston, Massachusetts, United States of America

## Abstract

Activity patterns and complex functions of the brain rely on the characteristic communication network formed by axonal fiber networks, but how many axons actually connect different brain regions? This Primer explores a study in PLOS Biology which finds that most areas of the human cerebral cortex are linked by an astoundingly small number of fibers.

## Teaser

Brain functions rely on the communication network formed by axonal fibers. However, the number of axons connecting different brain regions is unknown. A study in PLoS Biology addresses this question and finds that most areas of the human cerebral cortex are linked by an astoundingly small number of fibers.

In recent years, network neuroscience has provided a new perspective of how brain function emerges from the communication among distributed, specialized regions in the brain. This framework explains patterns of neural activity and brain functions on the basis of the structural connections among brain regions. The structural connection scaffold has been uncovered systematically by histological approaches in non-human primate models [1] as well as by non-invasive approaches, such as diffusion imaging tractography, in the human brain [2]. The structural connectome was shown to possess a characteristic, non-random organization that strongly shapes activity patterns and functional connectivity of the brain [3]. Therefore, long-range axonal projections are assumed to play an essential role in exchanging signals among remote regions.

Amazingly, however, while the general existence or the relative strength of pathways connecting regions of the human brain can be well estimated, the actual numbers of neurons linking one region to others are still uncertain. This uncertainty is due to methodological limitations. For example, the number of streamlines inferred in diffusion tractography varies with the voxel resolution of the imaging data, and the number of stained projection neurons in histological studies depends on the amount of injected tracer, so that current approaches only deliver estimates of relative, but not absolute connectivity.

**Fig 1:**
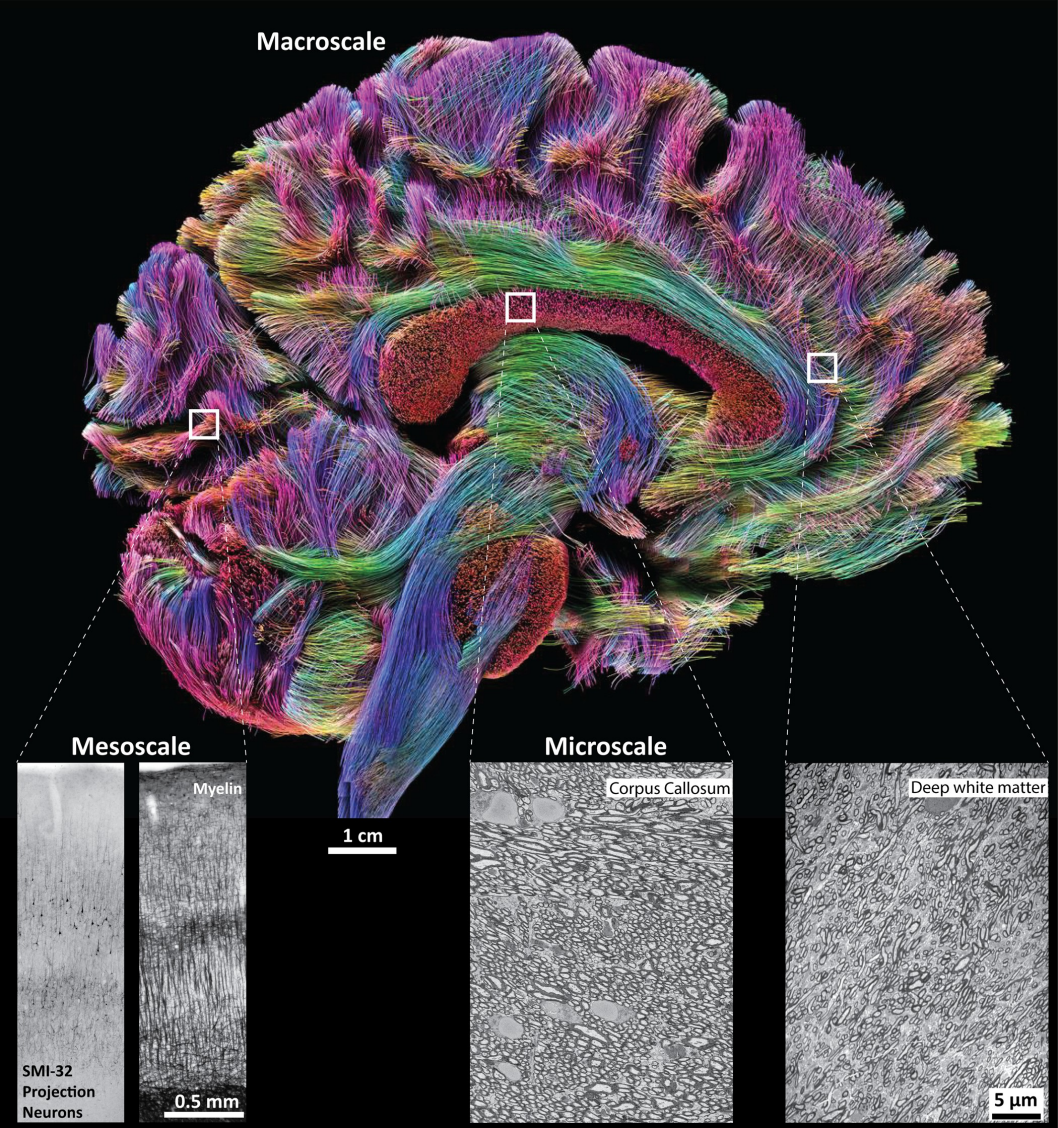
Integrating human brain connectivity at the macro, meso and micro scale. MR diffusion tractography is a widely used non-invasive method for the macroscopic estimation of anatomical connections in the living human brain (macroscale, top image). Higher resolution, albeit invasive, tractography approaches can only be used in animal model systems or with post-mortem human brain tissue to study projection neurons and axon bundles at the cellular level (mesoscale, bottom left panels of cortical columns in visual cortex labelled with non-phosphorylated intermediate neurofilament protein SMI-32 or with the Gallyas myelin stain). To study individual axons and synaptic interactions between connected neurons and areas one needs to use ultra-high resolution approaches and visualize brain tissue under the electron microscope (microscale, two bottom right panels of myelinated axons in the corpus callosum and deep white matter below the cingulate cortex). By comparing macroscopic counts of MR diffusion streamlines with microscopic counts of axons passing through the corpus callosum, Rosen and Halgren [4] derived a conversion factor that can be applied to tractography data in order to estimate the absolute number of axons in a given tract. However, caution needs to be applied when translating the factor from the well-ordered callosal connections to more heterogeneous non-callosal fiber tracts, such as shown at the bottom right. The calculation suggests that, despite the massive communication system formed by the white matter of the human brain, the absolute number of projections linking any two cortical areas on average is quite small. The finding prompts questions of the modes and neural mechanisms of communication between human cortical areas. Main image: courtesy of the USC Mark and Mary Stevens Neuroimaging and Informatics Institute (www.ini.usc.edu); insets: unpublished data by B. Zikopoulos.

This problem was addressed by Rosen and Halgren [4] who compared post-mortem axon density in the human corpus callosum with estimates of callosal streamline density from non-invasive diffusion tractography (Fig 1). Such comparison provides a conversion factor of streamlines to axons which can be applied to the streamline connections between any two brain regions, providing an estimate of the absolute number of axons running between them. This number turns out to be astoundingly small, on average just about 6,000 axons connecting any two areas in the same cortical hemisphere. The average number of connections between areas in different hemispheres is even smaller, below 1,500 axons. While previous studies have hinted that connectivity between some areas could be sparse [1], the overall sparsity of cortical connections implied by the present study still comes as a surprise. It is as if a traffic system presumed to consist of multilane highways running between most brain areas in fact consists of just a few precarious footpaths.

The small number of projections can be seen against the background of the number of neurons under one square millimeter of cortical surface, which is in the order of 60,000 neurons [5]. About 80% of these neurons are pyramidal projection neurons with axons that travel in the white matter [5], Fig 1. However, since most axons in the white matter participate in short-range connections, only about 10% of pyramidal neurons may project to remote areas [4,6]. Given that cortical areas are linked with a multitude of other areas and subcortical regions, it becomes clear that fiber paths between any two cortical areas are formed by just a small percentage of their projection neurons.

To give a functional example, Rosen and Halgren [4] looked at a structural pathway assumed to be essential for language function, and found that just 1–5% of the axons in the middle section of the arcuate and superior longitudinal fasciculi directly connect Broca’s and Wernicke’s areas. This result echoes a similar calculation of the number of thalamic projections terminating on the receiving layer of primary visual cortex in the primate brain, which found that only about 5% of the synapses in the visual cortex were coming from the lateral geniculate nucleus of the thalamus [7]. These findings generally suggest a predominant amount of recurrence of local cortical connections, combined with the strong amplification of signals coming from the sensory periphery or from remote cortical and subcortical areas.

Despite the generally very sparse connectivity, there do exist some substantial connections, highlighting the factors that underlie the existence of such highways among a majority of byways. Distance has been indicated as one of these factors, while another is the cytoarchitectonic similarity of connected regions, according to the structural model of connections [8], which also specifies other fundamental aspects of cortico- cortical projections, such as their laminar origin and termination patterns. Based on these principles of cortical network organization, we know that cortical areas tend to be connected primarily with other areas that are relatively similar in cellular architecture. Most of these similar areas with strong connections happen to be nearby, but some can be quite distant, for example in the case of the relatively strong long-range connections between architecturally similar lateral prefrontal and parietal cortices [8].

But do these principles imply that cortical signals only propagate between strongly connected adjacent or architectonically similar areas? What then is the functional role and significance of the majority of sparse long-distance projections in the human cortex? More specifically, what are the mechanisms by which signals may become amplified even though they are traveling along just a few projection axons? Models by which signals simply diffuse across the global network do not appear plausible given the sparsity of most long-distance projections relative to the massive number of local connections. By contrast, potential mechanisms through which long-range projection signals could be amplified include particularly large projection neurons [9], the formation of multiple or highly effective synapses [10], the targeting of cellular substructures close to the nerve cell body that are particularly responsive, as well as the subsequent amplification of incoming signals by local cortical circuitry.

The finding of sparse connectivity crucially hinges on the inferred conversion factor of streamlines to axons. Therefore, Rosen and Halgren [4] were concerned with getting this conversion factor right and used control computations for several constraints on the reliability of the conversion to verify that the factor is at least correct within an order of magnitude. There are still a number of caveats, particularly concerning whether the relations established for the corpus callosum translate also to other fiber systems within each cortical hemisphere (Fig 1), which should be tackled in future studies.

Future comparative investigations may also address if the connection sparsity found here is particularly characteristic of the human brain, or if large brains of other animals possess similar sparsity. This question is relevant in the context of functional interpretations of sparsity as a basis for the segregation and stabilization of signals in the human brain [11].

The skillful integration of cortical scales demonstrated by the present study [4] may shape a re-thinking of models of cortical activity and function, and may be particularly helpful for the fine-tuning of large-scale models of the brain that are based on representing the exact numbers of local and long-range cortical connections [12].
